# Dual-Use Research Debates and Public Health: Better Integration Would Do No Harm

**DOI:** 10.3389/fpubh.2014.00114

**Published:** 2014-09-12

**Authors:** Jonathan E. Suk, Cornelius Bartels, Eeva Broberg, Marc J. Struelens, Amanda J. Ozin

**Affiliations:** ^1^European Centre for Disease Prevention and Control, Stockholm, Sweden

**Keywords:** laboratory biosafety, Public Health, infectious diseases, dual-use research of concern, biosecurity, laboratory biorisk management, risk assessments

## Introduction

The rapid pace of discovery in the life-sciences can have profound implications for public health, and the focus of much deliberation in recent years has been on how best to ensure that they are positive and not negative. A key focus of debate has been on dual-use research of concern (DURC), which has been defined as life-science research that “could be directly misapplied to pose a significant threat with broad potential consequences to public health and safety, agricultural crops, and other plants, animals, the environment, materiel, or national security (1).” Debates such as the one that surrounded gain-of-function (GOF) research on avian influenza have led to many existential questions about contemporary life-science research, including whether or not such research should even be conducted in the first place, what viable alternative experimental approaches exist, if or how the findings should be made public, and how – or whether – such research can be governed (2–6).

Responding to these questions at a policy level necessarily involve a broader sphere of actors than life scientists alone as they have potential ramifications in different sectors and for society-at-large. Often, it is the security and research communities that have been at the frontline of such debates and driving policy. The public health community tends to enter the fray at later stages, such as after the completion of “concerning” research, at which point it is asked to either facilitate discussion or comment on the potential public health risks and benefits of research (7, 8). By that stage, public health organizations risk being viewed of as a partisan supporter of dual-use research (9).

In this paper, we demonstrate how the public health sector could more substantially contribute to the debate, guide policy decisions, and promote actions along all phases of the research life-cycle. Before doing so, we articulate the key aspects of the dual-use debate as they are relevant to public health.

## “Do No Harm”: Public Health and Dual-Use Research

Medical research is intended to promote the health of humans at an individual and a population level along a set of ethical principles laid down in the Declaration of Helsinki^1^ and overarching professional ethos in health care to “above all, do no harm” – *primum non-nocere*. What complicates matters with regards to the dual-use research in the area of health is that harm could potentially be done by both promoting and preventing research. On the one hand, research fuels innovation in new medicines, vaccines, and diagnostics, which are fundamental components of public health intervention strategies. The fine balance required for tightening up the regulation of life-science research may, in some instances, lead to some undesirable consequences, such as greater barriers and costs for doing research on listed agents^2^ (10). On the other hand, promoting DURC increases the possibility of malevolent use, through providing the information or material that would help state or non-state actors to develop agents for a biological attack. After pathogens are modified or created, responsibilities are created for their physical containment; the issue is thus one of both laboratory biosafety and biosecurity. Even more problematically, once DURC experiments are published, it is nearly impossible to control access to information. In this sense discussions about the risks and benefits of DURC nearly always occur too late – ideally, they should occur before and not after the research has been conducted.

The wide spectrum of potentially problematic issues related to dual-use research necessitates multi-stakeholder engagement including life-science researchers, research funders, regulators, the scientific media, ethicists, social scientists, and security communities. In addition, given the potential societal implications of the topic, greater transparency, and public engagement surrounding dual-use debates is required (11).

There are numerous highly debated issues that have surrounded dual-use research. At the broadest level has been the question of what sorts of life-science research should be conducted, and what constitutes DURC (12, 13). Regulatory measures, such as security clearances for researchers and export controls (10, 14) as well as other risk mitigation measures (1) have been contemplated alongside self-governance measures such as raised awareness and codes of conduct and continued professional development for life scientists (15–18). Furthermore, much attention has focused on whether or how results should be published (6, 19) and how practical measures, such as enhanced laboratory biorisk management processes along (global) standards, can be achieved (7, 20).

From the perspective of public health, these are all important discussions, each aimed at identifying and mitigating potential harm. However, in order to ensure better integration of public health aspects into future policies and actions, a few other key issues must be addressed. One relates to the way in which risk assessments of specific dual-use issues could be strengthened. Another builds upon previous arguments for managing dual-use research by taking into account risk-based approaches and biorisk (biosafety and biosecurity) management in all areas of implementation of the research cycle (7, 21). Addressing all of these public health relevant aspects could offer a next step in structuring the dual-use debate into options to mitigate harm through joint action between the research, security, and policy making communities.

## Reducing Information Asymmetries in Risk Assessments

A striking feature of the dual-use debate is that the benefits to public health are often invoked, regardless of the extent to which actors in the public health sector have been consulted. This is particularly ironic given that one core area of public health expertise is the undertaking of risk assessments. For example, one of the rationales for moving beyond the moratorium on publishing A(H5N1) transmission studies was they were “essential for pandemic preparedness” (4). This statement was made after an expert consultation organized by the World Health Organisation (WHO) in February 2012 (7). However, it is important to note that the WHO consultation occurred after the research had been completed and this consultation has been criticized for the absence of a broad range of expertise^3^. It should furthermore be noted that although the obtained study results are of scientific value, some have pointed out that incorporating detailed sequencing studies on the viral samples from infected patients is, for many countries, likely not the most feasible or effective way of strengthening global pandemic preparedness in the short term (19, 22). Previous research has noted that there are, globally, key gaps in viral sample collection and analysis (23). In addition, although the longer-term benefits may well be substantial, translating the findings from advanced life-science research into tangible and usable products and knowledge can take years, if not decades.

It is essential to keep in mind that advanced research is not always predictable. Discoveries can be serendipitous and researchers themselves may not always be able to predict what sort of outcomes certain experiments might lead to. The point here is not to argue against advanced life-science research but to caution against the creation of overly ambitious expectations, particularly, if these will be the basis for risk-benefit discussions about dual-use research. Scholars of science and innovation, for example, have long observed that the creation of sometimes overly optimistic expectations about the future benefits of research helps to secure funding and lower regulatory hurdles (24, 25). Similarly, it has been pointed out that large funding bodies bring particular interests and institutional cultures to the dual-use debate, one which tends to emphasize the positive benefits of such research (9). In the specific example of GOF research on influenza viruses, it is notable that at least a few prominent scientists believed that the benefits had been overstated (22, 26, 27). One way of mitigating the development of over-expectations for research is to ensure balanced debate and doing so could be achieved by comprehensively including a broader range of perspectives, including public health, in both pre- and post-experimental discussions (11, 26).

There is another important information asymmetry on the other side of the spectrum in debates about dual-use research. This relates to the intents and capabilities of would-be bioterrorists and how this affects risk assessments (28). Presumably, the principle reason for worrying about publishing experimental protocols or genomic information relates to the concern that rogue scientists could replicate the results with malevolent aims. Given the rapid advances in life-science research, the advent of synthetic genomics and the numerous social issues that it raises (29, 30), and the declining costs of doing research, it would seem quite reasonable indeed that a wider range of actors might be able to replicate advanced research. Yet this assumption may appear to overstate the ease with which advanced research can be undertaken, which relies not only on information and materials but on experimental know-how and tacit knowledge (31). The recent history of actual bioterrorist events as well as the findings from a risk analysis appears to support this claim (32). This is a key point. Overstating – or understating – the capabilities and intents of bioterrorists can affect the perception of the “riskiness” of research. Here, the public health sector needs to raise and reiterate the importance of this question. The aim should be to ensure risk assessments integrate the best available information from a variety of sectors, meaning that life scientists, regulators, ethicists, public health actors, *and* the security and intelligence communities will need to become more adept at and comfortable with exchanging information and ideas. This has not always been the case.

## Addressing all Phases of the Research Cycle

The WHO advocated managing dual-use risks by taking account of all stages of the research cycle, which is an approach that we would like to briefly elaborate upon here (7). Public health activities already encompass many of these phases, and thus existing expertise could be harnessed to ensure a broad and comprehensive public health engagement with dual-use research (Table 1). Following the discussion above, in the “pre-research” phase, the public health sector could contribute to discussions about the possible risks and benefits of research through consultation with research funding bodies, scientists, and institutional review boards. In addition, good laboratory practice means following ethical and legal guidelines for conducting research on infectious diseases, and for ensuring that robust biorisk management systems are in place. Some European public health institutes have developed tools to facilitate this, such as the Dutch Biosecurity Self-Scan Toolkit^4^ or the German development of codes of conduct for potential dual-use research^5^.

**Table 1:** **Examples of public health risk mitigation strategies along the phases of the research cycle**.

Research phase	Examples of public health risk assessment and mitigation measures
Pre-research	Advocate compliance with international obligations and treaties including:
	• Biological and toxin weapons convention
	• National legislation in place and oversight bodies aligned with EU regulations
	• Assessment of public health benefits versus the risk of DURC
	• Harmonized and updated ethics/biosecurity protocols
	Promote laboratory biorisk management system according to good practices and standards along the lines of CEN15793:2011 and WHO guidelines in biosafety and biosecurity. This would include:
	• Plan experimental needs according to risk assessments
	• Ensure availability of appropriate laboratory facilities
	• Continuing education of life scientists
	• Ensure researchers have the necessary security clearance
During research	Promote laboratory biorisk management system according to good practices and standards along the lines of CEN15793:2011 and WHO guidelines in biosafety and biosecurity. This would include:
	• Ensuring laboratory biosafety standard operating procedures (SOPs) for DURC occurring at research institutes – i.e., responsible biosafety officer role, appropriate facilities, well-trained staff, security clearance of scientists, appropriate facility oversight, well-trained staff, etc.
	• Reporting promptly any accidents or laboratory acquired infections to the defined authority in the SOPs
Post-research	Support discussion of the public health importance of findings and how the knowledge can support future public health programs/actions
All phases	Advocate for overall public health system capabilities such as:
	• Sufficient laboratory capacity for timely and reliable detection of infectious disease health threats
	• Harmonized biorisk management practices and strengthened investments in supportive research to address any gaps in practice
	• Education programs and continued professional development to build a culture of scientific responsibility
	• Ensuring public health perspectives in dealing with policy developments for DURC
	• Public health contribution to guide research priorities
	• Building and maintaining relationships with stakeholders (e.g., research funding, research, science publishing, and security communities)

During research, biorisk and biosecurity managers should communicate with researchers about emerging findings, and should regularly monitor the adherence to laboratory biosafety and biosecurity standards for research deemed potentially “risky.” It is essential to remember that all laboratory research carries a risk. The public health consequences of laboratory accidents are quite serious, particularly if the possibility exists that laboratory-infected workers expose the general public, as nearly happened with SARS (33). Early communication with public health agencies about findings likely to generate particular attention could also occur during this phase.

Post-research, a clear discussion on the potential public health benefits should be incorporated into risk analyses concerning the implications and publication of findings. Existing protocols for the physical containment of experimental materials should be reviewed and, if necessary, strengthened should DURC be approved. The recent misplacement of samples of SARS, anthrax and smallpox demonstrates the continued importance of maintaining high biosafety and biosecurity standards for storing physical specimens^6^^,^^7^.

During all phases of the research cycle, the public health sector works to strengthen its core functions such as surveillance, preparedness, prevention, response, risk communication, and training. In regions where vast amounts of funding dedicated to potentially “risky” life-science research, the public health community should argue for a greater role in participating in research funding prioritization, and risk-benefit assessments. It could also make the case for strengthened and sustainable investments in biosafety, biosecurity, and core public health services: should a dangerous pathogen be released, whether intentionally or unintentionally, then strengthened general defenses against infectious diseases will be essential. Finally, but essentially, should the public health sector seek to develop the role of “honest broker” in dual-use discussions, then it will need to work seriously at fostering engagement with key stakeholders, such as security communities and ethicists in addition to life scientists so as to ensure a comprehensive “web of protection” (4). Hosting international meetings focused on bringing together scientists, the security community, public health workers, regulators and possibly even the public could be an initial way of engaging important sectors of society in the debate.

## Conclusion

Advances in life-science research are staggering with the advent of synthetic biology, the latest in a long line of technological breakthroughs. There is little to suggest that this pace of change will slow in the future and the increasingly global nature of science means that all countries have a stake in ensuring that research is conducted and disseminated responsibly. Thus far, debates about dual-use research have tended to invoke public health rationales but there is much room for improvement for ensuring that public health perspectives are fully integrated into discussions. This will be challenging, and mechanisms and fora for doing so will need to be created. Yet there is much to be gained from doing so, for the ethics behind the debate closely matches the public health ethos: *primum non-nocere*.

## Conflict of Interest Statement

The authors declare that the research was conducted in the absence of any commercial or financial relationships that could be construed as a potential conflict of interest.

## Footnotes

^1^http://www.wma.net/en/30publications/10policies/b3/, accessed June 7, 2014.

^2^http://www.australiagroup.net/en/human_animal_pathogens.html, accessed May 15, 2014

^3^http://www.psandman.com/col/WHO-H5N1.htm, accessed May 14, 2014

^4^http://www.biosecuritytoolkit.com/mainMenu.html;jsessionid=AAE30461C0ABD5A9A0D7BC33E91DBBD5, accessed May 20, 2014.

^5^http://www.rki.de/EN/Content/Institute/Dual_Use/code_of_conduct.html;jsessionid=A2FBBD63B5FE63F62EC4B84C0603C295.2_cid298?nn=4005636, accessed May 20, 2014.

^6^http://www.pasteur.fr/fr/institut-pasteur/presse/documents-presse/communique-presse-l-institut-pasteur, accessed May 20, 2014.

^7^http://www.cdc.gov/media/releases/2014/p0711-lab-safety.html, accessed August 4, 2014
